# Evaluation of SHM System Produced by Additive Manufacturing via Acoustic Emission and Other NDT Methods

**DOI:** 10.3390/s151026709

**Published:** 2015-10-21

**Authors:** Maria Strantza, Dimitrios G. Aggelis, Dieter de Baere, Patrick Guillaume, Danny van Hemelrijck

**Affiliations:** 1 Department of Mechanics of Materials and Constructions, Vrije Universiteit Brussel, Pleinlaan 2, Brussels 1050, Belgium; E-Mails: daggelis@vub.ac.be (D.G.A); danny.van.hemelrijck@vub.ac.be (D.V.); 2 Department of Mechanical Engineering, Vrije Universiteit Brussel, Pleinlaan 2, Brussels 1050, Belgium; E-Mails: dieter.de.baere@vub.ac.be (D.D.); patrick.guillaume@vub.ac.be (P.G.)

**Keywords:** acoustic emission, additive manufacturing, structural health monitoring, liquid penetrant inspection, radiography, eddy current

## Abstract

During the last decades, structural health monitoring (SHM) systems are used in order to detect damage in structures. We have developed a novel structural health monitoring approach, the so-called “effective structural health monitoring” (eSHM) system. The current SHM system is incorporated into a metallic structure by means of additive manufacturing (AM) and has the possibility to advance life safety and reduce direct operative costs. It operates based on a network of capillaries that are integrated into an AM structure. The internal pressure of the capillaries is continuously monitored by a pressure sensor. When a crack nucleates and reaches the capillary, the internal pressure changes signifying the existence of the flaw. The main objective of this paper is to evaluate the crack detection capacity of the eSHM system and crack location accuracy by means of various non-destructive testing (NDT) techniques. During this study, detailed acoustic emission (AE) analysis was applied in AM materials for the first time in order to investigate if phenomena like the Kaiser effect and waveform parameters used in conventional metals can offer valuable insight into the damage accumulation of the AM structure as well. Liquid penetrant inspection, eddy current and radiography were also used in order to confirm the fatigue damage and indicate the damage location on un-notched four-point bending AM metallic specimens with an integrated eSHM system. It is shown that the eSHM system in combination with NDT can provide correct information on the damage condition of additive manufactured metals.

## 1. Introduction

Optical inspection and a large number of non-destructive evaluation techniques can be inspiring for the development of Structural Health Monitoring (SHM) systems. In prior research, various SHM systems have been investigated in order to detect damage, which is a local phenomenon, by measuring the total response of a structure with the main research focus on damage identification in components [1]. Furthermore, the current research on SHM systems is particularly focused on and inspired by the biological nervous system, resulting in using various types of complicated sensor systems that try to mimic the living skin [2,3]. Nevertheless practical effectiveness, durability, and robustness of the SHM systems in real operating conditions remain a challenge. SHM’s continuous monitoring capability (especially during fatigue) has certain advantages when compared to periodic Non-Destructive Inspection (NDI) [4]. As stated by Speckmann *et al.* [5], SHM is an alternative approach of NDI for the inspection of the structural integrity of aircrafts and can be a major part of the prospective intelligent structures. SHM systems of the second and third generation will be used in order to obtain a novel approach to structural design that relies on lightweight structures. As a result, the SHM system should be an embedded part of a structure. 

Furthermore, additive manufacturing (AM) has been progressively gaining momentum during the past decade and can be included on the list of novel approaches to structural design. AM is material-efficient manufacturing method that enables the production of (near) net-shape products with the capability to create complex structures which cannot be produced by conventional production techniques [6,7]. AM is an emerging technology and can be tailored to the needs of building structures with embedded SHM systems, which is the main challenge for the SHM systems. In this respect, we developed a novel concept––the so-called “effective structural health monitoring” (eSHM) system. The current system uses one of the benefits and the greatest strengths of AM, which is creating complex and light structures. The main philosophy of the eSHM system is checking the absolute fluid pressure variations in a 3D network of capillaries or cavities that are integrated by AM techniques in the interior of a part. A sudden pressure change in the capillary indicates the presence of a crack between the outer surface and the capillary. More details on the eSHM system development can be found in [8]. The pressure inside the capillary is initially significantly lower (or higher) than the ambient pressure. In both cases, the absolute values of the detection signals are very comparable for the same delta pressure (ΔP). In the case of positive pressure, the ΔP with the environment can be made larger than 1 bar, which results an increased sensitivity. However, by increasing the pressure level above ambient pressure, the stress level at the inner surface also increases which leads to a smaller fatigue strength of the system. Nevertheless, for applied pressure levels of approximately 1 bar these effects are rather small. The novel eSHM system can monitor a complete structure with a single pressure sensor to observe the pressure in the capillary network. The capillary or cavities are integrated in the metallic structure by means of AM and as such mimic the biological nervous system. The size of the capillaries can currently vary between 1 and 3 mm in diameter. The response of the structure with and without capillary is demonstrated in [9] where the eSHM system has proven its effectiveness. The eSHM system does not influence the crack initiation behavior and as a result the fatigue life as it is shown in [10]. This is concluded from an experimental and numerical analysis of various capillary locations and their impact on the structure. It is of high importance to mention that this is not the first time that the current measurement principle is used. Those capillaries (or cavities or elongated holes) have already proven their effectiveness in similar applications. Straight elongated capillaries were used in previous applications for crack detection in rotorcrafts [11]. There are also a few patents in similar applications where elongated capillaries are used for monitoring without weakening the part [12,13,14]. Failures of various materials that are used in aerospace are detected and inspected by means of non-destructive testing techniques (NDT). There are many NDT methods that can be used to examine a material, such as visual inspection, eddy current, ultrasonic inspection, acoustic emission, radiography, *etc.* [15]. NDT techniques are also widely used in metals. Electromagnetic methods like eddy current (EC) or radiographic methods introduce electromagnetic waves into the material in order to evaluate the material [16]. EC allows crack detection in a wide range of metals. It is used in order to locate surface and subsurface defects (few mm below the surface), to determine the hardness of metals which have been subjected to heat treatment [16] and also to provide information on the residual stress field close to the surface [17]. The main principle of the EC technique is based on electromagnetic induction where the interaction between a magnetic source (produced by the probe) and the subject material is observed in order to detect a discontinuity or a defect. Radiography is used as a non-destructive technique providing information of defects and the internal state of a metallic sample [18]. Radiography requires the projection and penetration of radiation energy on and through an inspected material. The radiation energy is absorbed homogenously by the material, except in the regions where thickness or density variations arise. The energy that passes through is captured by a sensing medium (film) in the form of an image of the interior of the specimen. Liquid penetrant (LP) inspection can also be used successfully in the inspection of metals, either with fluorescent or non-fluorescent dyes. Furthermore, NDT and evaluation methods such as X-rays, neutron diffraction, magnetic Barkhausen noise (MBN), EC, *etc.* can be also used in order to characterize materials and perform residual stress evaluation [19,20,21,22]. Some of those techniques (MBN and EC) are mainly influenced by the magnetic properties of magnetic materials [23]. More specifically, MBN is rather sensitive to microstructure and applied stresses [24] while eddy current signals are sensitive to the magnetic permeability of materials, which depends on the stresses [20]. Both techniques are characterized by reliability and no special specimen preparation is required prior to application. Furthermore, eddy current thermography, which is a combination of eddy current and IR thermography, was also used in the health monitoring of metals [25].

**Figure 1:**
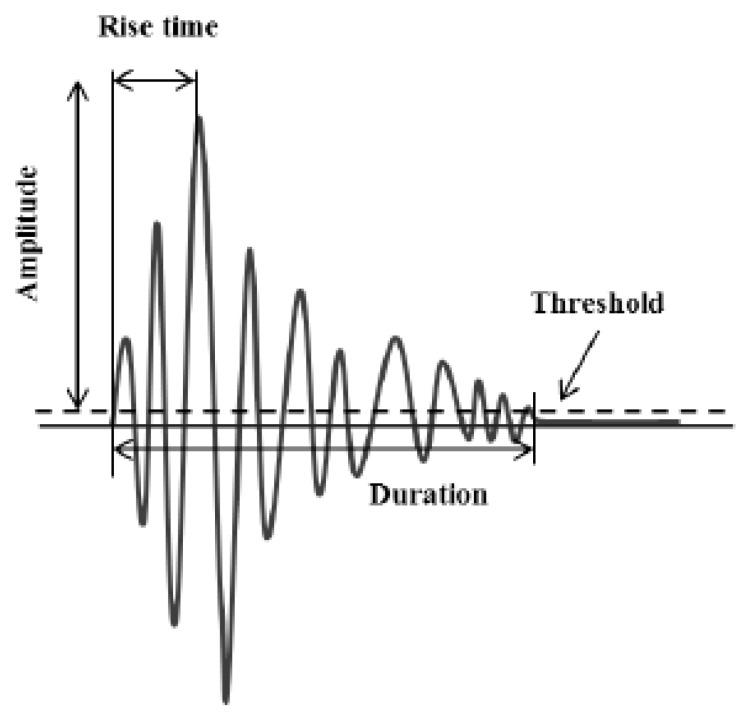
Typical acoustic emission signal with main waveform parameters.

Additionally, acoustic emission (AE) is another NDT technique that uses elastic waves that are emitted in a medium due to crack nucleation or propagation. These elastic waves can be captured by suitable piezoelectric sensors on the surface of a specimen [26]. Besides the number of emitted acoustic signals, which may provide information on damage accumulation, other qualitative waveform parameters can be also significant. Parameters like maximum amplitude, energy, rise time (RT) and duration (DUR) can provide equally important information. Figure 1 shows a typical AE waveform with its basic features. AE as a technique has been extensively used in damage characterization in metals [27,28,29,30], rocks [31] and ceramics [32] among other materials while structural components like wind turbine blades [33] were also evaluated. Furthermore, in cyclic loading histories other important effects like the well-established “Kaiser effect” and “Felicity effect” should also be taken into account as essential damage accumulation indicators. In the case of the Kaiser effect, no AE is recorded until the previous maximum load has been overpassed while reloading an intact specimen [31,34,35]. On the other hand, AE will be recorded in cases of severe damage even at much lower loads than the previously applied [32]. In those cases, the ratio of the load when AE takes place and the previous maximum load is called Felicity ratio. According to the literature [36,37] Felicity ratio 1 or larger shows no damage since the last AE inspection. However, a decrease in the Felicity ratio value might be associated with cumulative damage.

The main objective of the current article is the evaluation of the damage detection capability of the eSHM system. This evaluation will be performed by NDT inspection in stress-free conditions and during static load conditions. Prior to this work, previous research on the response of the eSHM system during four-point bending fatigue testing was conducted [38] and cracks nucleated by this process will be assessed in this paper. Before the eSHM system can be implemented in real structures, its results concerning sensitivity to damage initiation and propagation should be validated. To do so, we applied different NDT techniques (liquid penetrant, eddy current, radiography, and AE) to monitor crack development and location, and their results are discussed in relation to the eSHM. To the authors’ best knowledge, this is the first effort to monitor damage in AM metallic components of SS 316L and Ti6Al4V by the AE technique. The major contribution of the study lies in two fields. One is the aforementioned evaluation of the adequate function of the eSHM system, *i.e.*, confirming the existence of a crack that was detected by capillary pressure change using a combination of monitoring techniques and demonstrating that these NDT techniques also work on AM Ti6Al4V and AM SS 316L. The second major contribution concerns to the AE and the possibility of this passive technique to complement the data of the system. AE produces numerous signals in several SHM cases, making difficult to discriminate between the relevant and non-relevant emissions (noise, signals outside the gauge length of interest). The indication of the eSHM system may help focus on the AE activity from the moment the crack is detected. 

The second section of the article describes the test specimen with the integrated eSHM system, the loading procedure during static loading and the experimental details of the NDT techniques. The results are presented in the third section. The first part of the third section starts with the results obtained with NDT methods in stress-free conditions. In the second part of the third section, the LPI was performed under loaded conditions and the correlation of the pressure measurements of the system will be discussed. In the last part of the third section, the applicability of AE for AM components is presented and analyzed focusing on the standard AE parameters and the localization capability of AE. Remarks and conclusions based on the results are given in Section 4. It is shown that the aforementioned NDT techniques can be successfully used in this innovative class of materials. Furthermore, AE parameters like rise time and amplitude can provide valuable information on the damage accumulation while well-known trends and phenomena like the Kaiser effect also occur in this innovative class of materials.

## 2. Experimental Section

As previously mentioned, the specimens for this study were produced by additive manufacturing. In total, there were two Ti6Al4V produced by selective laser melting (SLM) and two AISI 316L produced by laser metal deposition (LMD). SLM and LMD are additive manufacturing processes and they both use the energy of a laser beam in order to bind metal powder particles. They mainly differ in the way the powders are deposited [39]. 

**Figure 2:**
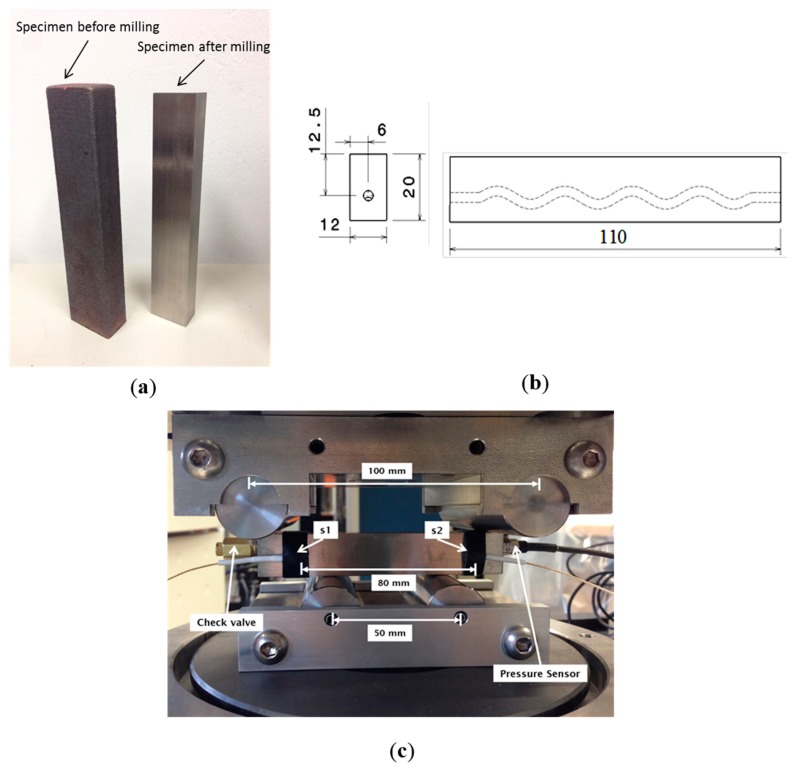
(**a**) Specimens produced by laser metal deposition (LMD) before and after milling; (**b**) four-point bending specimen, 2D side and front views; and (**c**) four-point bending setup with the AE and the pressure sensors placed on the specimen.

All the specimens were built with an integrated eSHM system by means of a 3D sinusoidal shape of capillary, which was built in the course of AM. The integrated capillary had a diameter of 3 mm in the LMD samples (AISI 316L) and 1 mm in the SLM samples (Ti6Al4V). The sinusoidal shape had an amplitude of 2 mm and a period of 25.7 mm. The current design lies on a previous study, where the main objective was to prove that the integrated capillary had no negative influence on the crack initiation during fatigue. The sinusoidal shape is also linked with the ability of AM to create complex structures. The locations with the highest likelihood for crack initiation from the capillary were situated on the circular cross sections that were furthest away from the specimens’ neutral axis. In the current specimens, the moment of inertia alters along the capillary. This is associated with the fact that the circular cross section of the capillary from the neutral axis varied. The further the cross section from the neutral axis, the lower the moment of inertia and the higher the nominal stress and the stress at the capillary becomes. More details on the design strategy can be found in the paper of Strantza *et al.* [9]. After production the specimens were milled to the final dimensions shown in Figure 2b. A specimen before and after milling is also depicted in Figure 2a.

Prior to this study, the specimens were subjected to four-point-bending fatigue testing until damage was detected by the eSHM system. In order to locate the crack, NDT techniques such as LP, EC and radiography were applied. For the current study, the equipment that was used for radiography was of the Baltospot 200 kV type. The LPI level 2 and level 3 in unloaded conditions (ARDROX fluorescent penetrants 9704 and 9705) were applied. In order to locate the cracks by eddy current, a NORTEC 500 Series eddy current flaw detector from Olympus was chosen. It is known that in order to detect surface cracks in low conductivity metals like stainless steel and titanium, higher frequencies on the probe are required. In that study a pencil probe of 2 MHz (HGain 60 dB and VGain 70 dB) was chosen. Prior to the inspection, a sound reference specimen was used to balance the instrument and to set the axis.

The specimens with cracks initiated by fatigue, as mentioned in the previous paragraph, were also subjected to a static four-point bending loading. This test setup produces a uniform moment with no shear force between the two inner loading rollers in the specimen. Initially, a pressure transducer (Kulite XTL-123C-190M-1,7-BAR-A) was installed at one side and a check valve (Clippard MCV-1-M5) at the other side. Before the installation of the check valve, a thread sealant (Loctite 577) was applied on the first few threads of the valve as an adhesive between the specimen and the check valve. After sealing, the capillary in the specimen was subjected under-pressure of 0.5 bar. As the final step, a stop (Clippard 11755-M5-PKG) was installed on the check valve as a supplementary blocking. The loading for each specimen was increased gradually in steps of 5 kN starting from zero up to the maximum load of the fatigue test. Crack detection by means of the eSHM system occured in both LMD samples at the fatigue load level of 34 kN (maximum tensile stress 530 MPa). In the first sample of SLM, the detection took place at 20 kN (maximum tensile stress 322 MPa), and in the second SLM sample, at the load level of 36 kN (maximum tensile stress 570 MPa).

Concurrently with the static loading procedure, the specimens were monitored by the pressure transducer and two AE broadband transducers (Figure 2c). The acoustic emission sensors were also placed between the supports at a distance of 80 mm. The sensors were of “pico” type of Mistras Holdings having a broadband response and peak frequency at 450 kHz. The signals were pre-amplified by 40 dB and recorded by the acquisition board with a sampling rate of 10 MHz (micro-II, 8 channels). The threshold was set at 40 dB and the acoustic coupling was improved by vaseline grease between the sensors and the metal specimen. The sensors were secured by means of tape during the loading. The analysis herein is based on the “AE events” rather than the total AE activity. An event is the source of AE and, in this case, consists of two signals received within a short window of time by both sensors. The location of the AE source can be determined between the sensors on the basis of the time delay between the two recorded signals, given that the pulse velocity in the medium is known. In the present case, localization was performed with a velocity of 5000 m/s. This velocity was measured by conducting pencil lead break tests. Analyzing only the events reduces the risk of noise contaminating the data, since it is always possible that friction in the supports or other irrelevant sources are recorded as individual hits. 

## 3. Results and Discussion

### 3.1. NDT in Stress-Free Conditions

After the crack was detected by means of pressure change in the eSHM system, the specimens were removed from the testing machine and subjected to LPI. In order to avoid false indications due to roughness, the specimens had been milled before testing (see Figure 2a). However, for this material and crack size, detection was not possible by means of LPI level 2. This may be associated with the fact that the crack is sufficiently closed in unloaded conditions. The indications of level 3 liquid penetrant were also not completely clear as they showed multiple indications apart from the real crack. LP tests were repeated under stress, as will be seen in the next section. 

**Figure 3:**
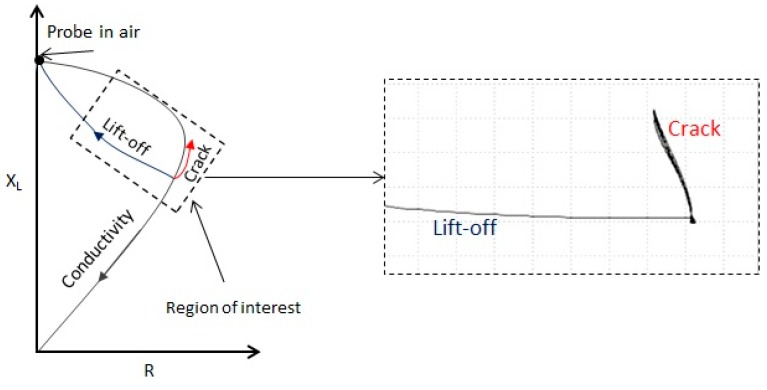
Eddy current (EC) signals from a normalized impedance plane diagram showing the defect detection in SLM2.

As far as EC is concerned, it is a technique that is used to examine rather small surface regions. The probe design and test parameters must be determined with a good understanding of the defect. The eddy current signal results are displayed in the normalized impedance plane of Figure 3. During the setup the coil resistance appears on the x-axis *versus* inductive reactance on the y-axis (see Figure 3). In order to detect cracks, it is more practical and common to adapt the phase in such a way that the lift-off (distance between probe and component) will be on the x-axis and the cracks will then appear on the y-axis by adapting the vertical amplification (depicted from SLM2 specimen in Figure 3 in the region of interest). By doing this, the phase between the lift-off and a crack will me more visible. The angle and amplitude change of the EC signal is the main principle for signal evaluation. The locations of the cracks were clearly detected in all the specimens along the length of 110 mm. In LMD1 and LMD 2 at 50 mm and 73 mm, respectively, while in SLM1 and SLM2 at 80 mm and 45 mm. 

**Figure 4:**
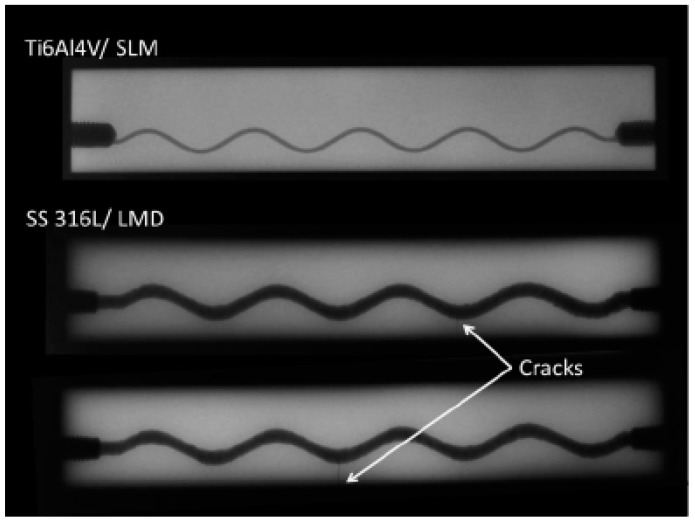
Radiographic images of the four-point bending specimens produced by selective laser melting (SLM) and laser metal deposition (LMD) after the crack detection of the effective structural health monitoring (eSHM) system.

Supplementary to EC and LPI, radiographic inspection was also performed on the specimens. In Figure 4 the radiographic images of the first sample of AISI SS316 and the two samples of Ti6Al4V are shown. The cracks are retrieved in both AISI 316L (see Figure 4) and are located on the lower part of the sinusoidal capillary on the positions that were also indicated by EC. However, the crack in Ti6Al4V could not be detected by radiographic images. The difficulties in detection may be associated with the angle between the crack and the radiation. If the radiation is not parallel with the defect, the defect may appear distorted, out of position, and less visible in the image.

### 3.2. NDT during Static Loading

**Figure 5:**
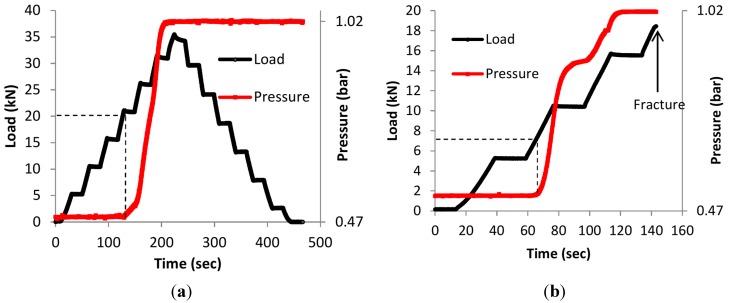
(**a**) Load history and time *vs.* time for the LMD1 sample; and (**b**) load history and pressure *vs.* time for the SLM1 specimens.

When the specimens are not under stress, the crack is closed so the internal pressure can be maintained. Therefore, prior to the static loading, pressure of approximately 0.5 bars was applied in the capillaries in order to monitor the crack opening again. During the test the specimens were monitored by AE. As previously mentioned, the loading was increased in steps of 5 kN until the maximum load of the fatigue test was reached, and then unloading was applied in steps of 5 kN. For the first two samples of LMD, the maximum load of the fatigue test was 34 kN, 20 kN for the SLM1 and 36 kN for SLM2. In Figure 5a the behavior of the pressure during the loading of LMD1 can be observed. At the load level of 20 kN a rise in the pressure is observed, where at 31 kN the pressure reaches the atmospheric pressure which is approximately 1.02 bar according to the pressure sensor. The pressure level of the SLM1 specimen, depicted in Figure 5b, indicates a pressure change at 7.5 kN where at 15.5 kN the pressure reaches the ambient pressure. It is obvious that for the SLM1 sample the pressure increase is noticeable quite early and a brittle fracture occurs below the maximum load level of crack detection, not allowing the controlled unloading to take place. This sudden failure was caused by porosity or unmelted regions of the SLM-processed material and led to an early failure. It is demonstrated in the literature that the porosity within the SLM samples has a radical effect on the failure and specifically on the fatigue behavior [40]. Pressure behavior similar to LMD1 was observed for the other two samples, which are not graphically represented. In all the cases, when the crack opens, the pressure inside the capillary starts to change to equalize with the atmospheric pressure. 

**Figure 6:**
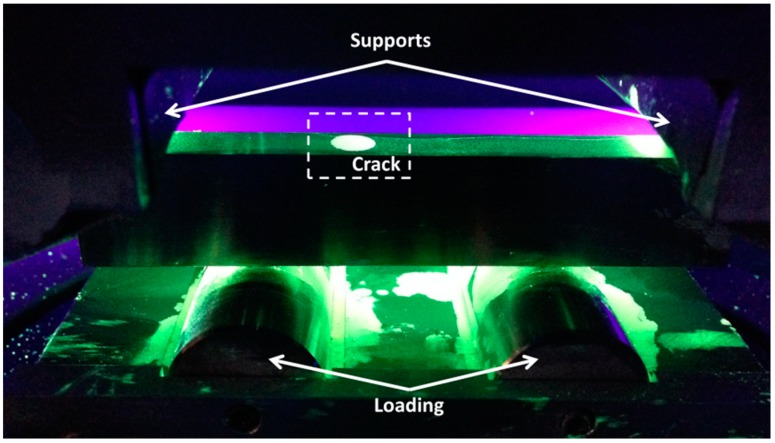
Crack location on additive manufactured samples by means of liquid penetrant inspection (LPI) level 2 under static loading.

After static loading, all specimens (except the one that failed during static loading) were subjected to another round of static loading on the load level of the crack opening. This new loading was important in order to confirm the presence of the crack by LP when it was open due to the tensile stresses. While, as mentioned, LP was not able to detect the cracks in stress-free conditions, since the crack was closed. At a certain load level of the crack opening, the specimens were coated by liquid penetrant. All the cracks were accurately located in all the specimens as is demonstrated for the LMD1 specimen in Figure 6. Those crack locations were the locations that were indicated earlier from EC in Section 3.1.

### 3.3. NDE by Means of Acoustic Emission (AE)

**Figure 7:**
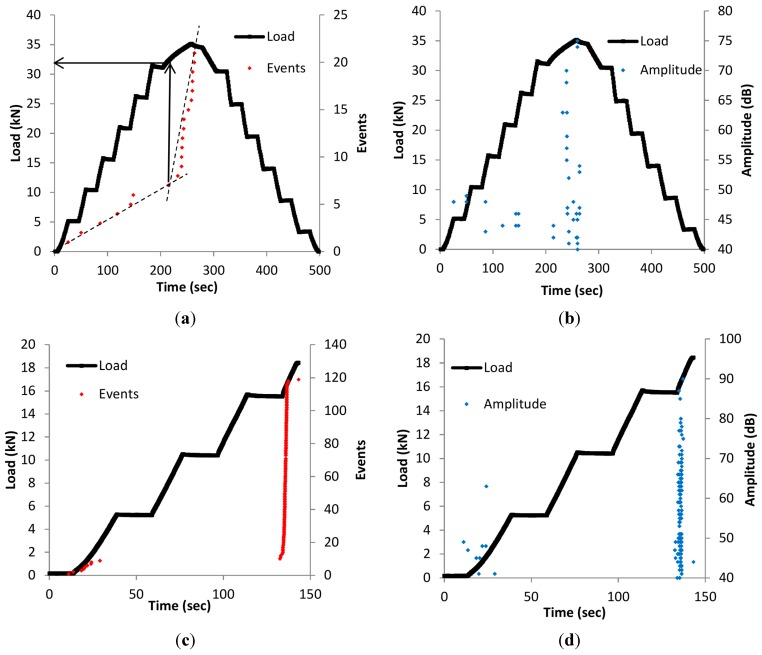
(**a**) Load history and number of events *vs.* time for the LMD2 sample; (**b**) load history and amplitude *vs.* time for LMD2 sample; (**c**) load history and number of events *vs.* time for the SLM1 sample; and (**d**) load history and amplitude *vs.* time for SLM1 sample.

In parallel with the static loading and the pressure behavior, AE technique was also used to monitor the sample behavior. In the current study the samples were already cracked so the study does not focus on crack initiation; however other mechanisms such as possible crack propagation and friction of the crack faces can be recorded. In Figure 7a the cumulative AE events and the load *vs.* time are depicted for the LMD2 sample. The event rate is moderate throughout the initial stages of loading and suddenly reaches a maximum value at the load of 34 kN which is the load level of the crack detection during fatigue loading according to the eSHM system. It is also characteristic that the cumulative number of AE events is seven until 32 kN and, up to 34 kN, the population has already increased to 21. Similar conclusions can be drawn from Figure 7b, where the amplitude and the load history *vs*. time are shown for the same specimen. Up to the load of 32 kN events do not exceed the amplitude of 50 dB. However, after that point there is also a population of signals above that level up to 75 dB. It is clearly observed that the AE hits with the highest amplitudes appear at the moment when the load reaches the maximum value of the load of the crack detection (between 30 and 34 kN), indicating possible crack propagation since the load reaches the previous maximum load applied on the specimen. 

In Figure 7c, the number of events is depicted for another sample, namely SLM1. As previously mentioned, SML1 failed during static loading after reaching the load of 18 kN. It is obvious that the total number of events (120) is much higher compared to the LMD2 sample, which was not totally fractured. Additionally, AE amplitude values (Figure 7d) have also increased significantly to 90 dB at the load level of the macroscopic fracture, while for the LMD2 specimen the value is limited to 75 dB. 

It has been mentioned that the absence of strong AE for low levels of load is in relation to the Kaiser effect that was described earlier in Section 1 [34]. In the case of LMD2 (Figure 7a), the activity during the static reloading started at 32 kN, 2 kN lower than the previously applied maximum load. This is reasonable since the material cannot be considered intact any more after fatigue cracking. This trend is related to the aforementioned Felicity effect where acoustic signals are emitted even at load levels lower than the previous maximum when the material is already damaged. The Felicity ratio is the ratio of the load where AE starts after the previous maximum load. In all the cases the Felicity ratios were calculated and the values varied between 0.898 and 0.943. These values confirm the damage accumulation in the additive manufacturing metal samples. It can be concluded that the Kaiser effect as well as Felicity ratio are valid for the specimens of this study and thus can be implemented in an NDT scheme of AM materials in general.

**Figure 8:**
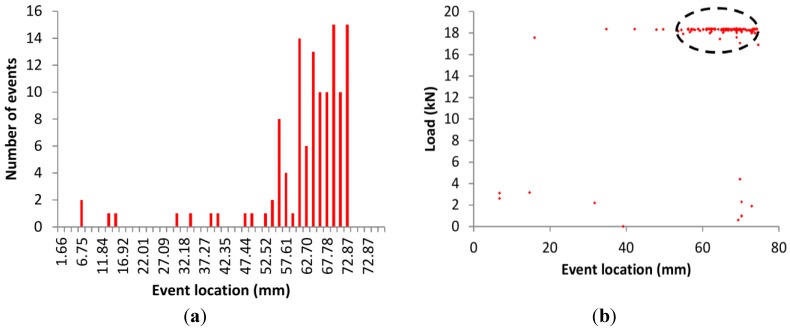
(**a**) Events *vs.* location for SLM1 specimen; and (**b**) load *vs*. event location for SLM1 sample.

In addition to the AE events history, the location of the events was also studied. Figure 8a shows the location distribution of AE events for SLM1 specimen. The event density is much higher for the position between 55 and 75 mm, where the actual fatigue crack was located (65 mm). The transient development of event location with load is seen in Figure 8b. Up to 5 kN load there are few distributed AE events (which at the location of the crack could indicate possible friction between the banks) while when the load approaches the previous maximum the crack starts to propagate and there are many events around the position of the crack, indicated by an ellipse. They are distributed within a zone of 20 mm rather than at a single location as can be concluded from Figure 8a,b. This is reasonable due to different factors of error, like the actual 3D geometry of the specimen (deviating from the applied “linear” localization method), short gauge length (80 mm) between finite diameter transducers (around 6 mm) and multiple reflections. It is important to mention that depending on the defect’s angle and size, NDT techniques such as AE, EC, radiography and LP can provide correct information on the location of a crack that is detected by the eSHM system. For example, in the case of SLM1, the AE events were localized at around 65 mm. The EC also indicated the crack at the position of 65 mm (which corresponds to 80 mm along the length of the specimen), where the actual fracture happened. On the other hand, the radiographic images and LP (level 2) in stress-free conditions could not provide a clear indication on the crack location for the SLM specimens as mentioned in Section 3.1. 

**Figure 9:**
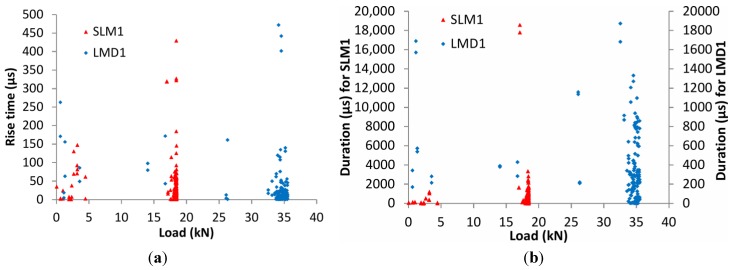
(**a**) Rise time *vs.* load history for SLM1 and LMD1 specimen; (**b**) duration *vs.* load history for SLM1 and LMD1 specimen.

In order to take into account the qualitative parameters of the waveforms rather than just their number, AE waveform parameters of duration and rise time were also analyzed. The rise time of the waveforms for the two sensors is depicted in Figure 9a for SLM1 and LMD1 samples. Each point represents the RT for each AE event during the static loading procedure. As previously mentioned, SLM1 specimen failed at 18 kN, while LMD1 followed the cycling loading up to 34 kN without fracture. It is obvious in case of both specimens that, when the load reaches its maximum value and crack propagation occurs, events with much higher rise time are emitted. The trend is very clear for SLM1, for which rise time was lower than 150 μs up to 15 kN, while at the end, values up to 430 μs were recorded. The same trend was repeated for the LMD1 specimen where the highest rise time values were noticed at the maximum load of approximately 35 kN, confirming that crack propagation in AM material can be detected by a change in waveform parameters. Similar results were also produced for the duration of the waveforms as depicted in Figure 9b. The duration for SLM1 sample was limited to 2000 μs below 15 kN, while for the maximum load, the duration increased to 18,000 μs. The same trend was also followed for LMD1 although the duration values were much lower (up to 1800 μs for the maximum load). Before the maximum load, the events with duration longer than 600 μs were scarce. However, when the load approached the maximum load the density of the events with high duration strongly increased.

## 4. Conclusions

In this study, the evaluation of the crack detection capability of a new SHM concept by means of various NDT methods was presented. The damaged metallic samples that were used for this study with the integrated eSHM system were inspected by a combination of techniques, namely LP (level 2 and 3), EC, radiography and AE. The techniques were complementarily used to evaluate the performance of the eSHM, and specifically to determine if the failure of the specimen indicated by means of the air pressure change in the eSHM capillary was confirmed by the NDT techniques. It was demonstrated that all four techniques can give correct information on the crack location depending on the defect’s angle and size. 

It was shown that the different techniques confirmed the existence of the crack, proving the reliability of the innovative SHM system and the suitability of the aforementioned NDT techniques to evaluate the structural integrity of AM components. This highlights the added value of those techniques in the localization capability of the system. This capability presents an advantage for industrial users who aim to validate the correct functionality of the system with the standard available NDT methodologies. More specifically, AE and EC successfully located the crack in AM components. From the moment that the crack is detected by the SHM system, the AE can also monitor its propagation. As far as LPI is concerned, although level 2 and level 3 did not provide the accurate crack locations in stress-free conditions, further investigations on including LP in the capillary should be conducted. If LP is placed in the capillary during testing, when a crack reaches the capillary and the system is activated, a portion of LP can also reach the surface of the specimen. The expected result is a better visualization (based on the LPI results in loaded condition) of the crack location in stress-free conditions by LPI since the LP will appear on the surface through the crack path and remain there until the inspection.

Furthermore, for the first time in literature AE was applied in AM components during static loading. Results show that AE follows the known trends as in other conventional metals. Specifically, the Kaiser effect and Felicity ratio are valid, while event location, despite the inherent difficulties due to small specimen size and multiple reflections, managed to indicate the actual zone of fracture. Additionally, AE parameters such as cumulative events, amplitude, rise time and duration are sensitive to damage propagation in order to lead to a warning against the final fracture occurrence. Since the suitability of classical AE is demonstrated, the next step is to use AE during fatigue in order to capture the actual nucleation of cracks which is expected to occur earlier than the detection of the eSHM system. Since AE is a very sensitive technique, the possibilities of detecting the crack even before it reaches the capillary should be exploited. In conclusion, the eSHM system can successfully detect damage on a metallic structure. Further investigations are conducted on the eSHM system in order to enable extra functionality of crack localization. While the eSHM system provides a unique crack detection capability, AE can complementary locate the crack and monitor the crack propagation of the detected crack.
